# Catheter localization in 3D ultrasound using voxel-of-interest-based ConvNets for cardiac intervention

**DOI:** 10.1007/s11548-019-01960-y

**Published:** 2019-04-09

**Authors:** Hongxu Yang, Caifeng Shan, Alexander F. Kolen, Peter H. N. de With

**Affiliations:** 1 Eindhoven University of Technology, Eindhoven, The Netherlands; 2 Philips Research, Eindhoven, The Netherlands

**Keywords:** Catheter localization, 3D ultrasound, Frangi pre-filtering, Convolutional neural network

## Abstract

**Purpose:**

Efficient image-based catheter localization in 3D US during cardiac interventions is highly desired, since it facilitates the operation procedure, reduces the patient risk and improves the outcome. Current image-based catheter localization methods are not efficient or accurate enough for real clinical use.

**Methods:**

We propose a catheter localization method for 3D cardiac ultrasound (US). The catheter candidate voxels are first pre-selected by the Frangi vesselness filter with adaptive thresholding, after which a triplanar-based ConvNet is applied to classify the remaining voxels as catheter or not. We propose a *Share-ConvNet* for 3D US, which reduces the computation complexity by sharing a single ConvNet for all orthogonal slices. To boost the performance of ConvNet, we also employ two-stage training with weighted cross-entropy. Using the classified voxels, the catheter is localized by a model fitting algorithm.

**Results:**

To validate our method, we have collected challenging ex vivo datasets. Extensive experiments show that the proposed method outperforms state-of-the-art methods and can localize the catheter with an average error of 2.1 mm in around 10 s per volume.

**Conclusion:**

Our method can automatically localize the cardiac catheter in challenging 3D cardiac US images. The efficiency and accuracy localization of the proposed method are considered promising for catheter detection and localization during clinical interventions.

## Introduction

Intervention therapies have been broadly applied to achieve a lower risk and shorter recovery period for patients, such as with cardiac catheterization for structural heart diseases. To clearly visualize and guide the catheter inside the body during the intervention, X-ray is typically used with a contrast agent to enhance the contrast. However, radiation, invisible tissue and lack of 3D information are key problems of X-ray imaging. Alternatively, 3D ultrasound imaging (US) offers richer spatial information on tissue and avoids radiation exposure, which makes it an attractive option for image-guided intervention. Nevertheless, localizing the catheter in US is often difficult because of the low-resolution and low-contrast US imaging. Therefore, automatic catheter localization in 3D US is highly desired for clinical practice.

### Related work

Medical instrument localization in the US image is achieved by classifying the US voxels. Uherčík et al. combined the image intensity with the Frangi filter response as a discriminating feature for voxel classification in needle localization [1, 2]. A recent study combined the Gabor features with Frangi features to localize the catheter in a phantom heart [3]. We previously used extended discriminating features within a multi-definition and multi-scale approach for catheter segmentation on ex vivo datasets [4]. However, these methods are less robust and less efficient when the US image has large variations in a complex anatomical environment. Recently, deep learning, e.g., convolutional neural networks (ConvNets), has shown significant performance improvement in medical image analysis [5]. For US imaging, the ConvNet has been commonly used to classify voxels into different categories. Two different approaches exist for this: voxel-based ConvNet and semantic-based ConvNet. The first approach classifies individual voxels one by one through the local information [6–9], while the semantic segmentation approach, i.e., fully convolutional networks (FCNs), predicts segmentation masks directly [10]. Although it has shown promising results by making use of the contextual information, the semantic segmentation approach requires a large number of training data and has high computational complexity.

**Fig. 1:**
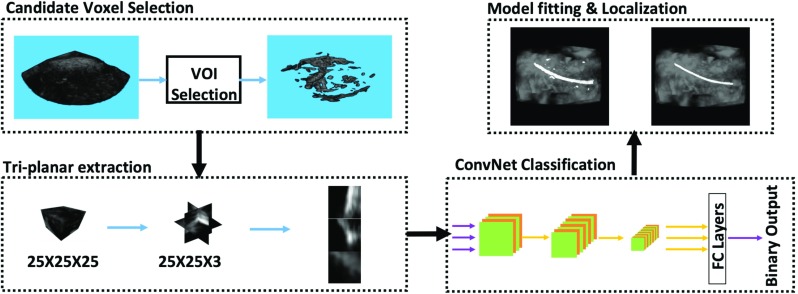
Diagram of catheter localization

###  Proposed approach

In this paper, we propose a catheter localization method for 3D cardiac ultrasound imaging. As depicted in Fig. 1, our method consists of three main steps. (1) Candidate voxel selection: a Frangi vesselness filter [1] first processes the input US image to coarsely select most of the candidate catheter voxels. The purpose of a voxel-of-interest (VOI) pre-selection procedure is to reduce the number of voxels to be processed by the ConvNet. To address the unstable response distribution in the Frangi-filtered image, resulting from variations in imaging conditions and catheter appearance [4], we introduce an adaptive thresholding method for the VOI selection, which allows to preserve the catheter voxels while omitting most non-catheter voxels. (2) Voxel classification through ConvNet: for each candidate voxel, a 3D neighborhood patch is extracted, and three orthogonal planes are extracted and processed by the ConvNet for voxel classification. In particular, we propose a simplified triplanar-based ConvNet, called *Share-ConvNet*, which reduces the computation complexity by sharing a single ConvNet for all orthogonal slices. We also combine two-stage training with a weighted loss function to improve the performance of the ConvNet. (3) Catheter localization: for the classified voxels, a cubic spline-based catheter model is fitted to localize the catheter.

Our contributions are threefold when compared to our preliminary work [9]. First, we employ a vesselness-based filter to coarsely select the candidate voxels to reduce the computation load for the ConvNet. With an adaptive thresholding strategy, most catheter voxels are preserved for further processing. Second, we proposed a Share-ConvNet for voxel classification in 3D US, which is in-depth compared with the existing methods. Third, we collect ex vivo datasets within challenging conditions, to thoroughly test the proposed method for catheter localization. The paper is structured as follows. Our approach is described in “Methods” section. The datasets and experimental results are presented in “Datasets and experimental results” section. Finally, “Conclusion” section concludes the paper with discussions.
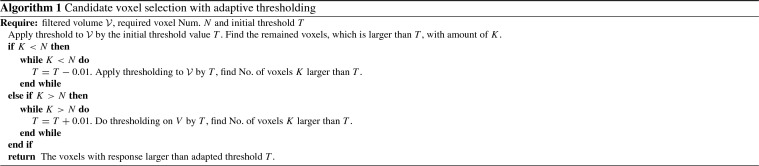


## Methods

### Pre-selection of candidate voxels

In our method, we use Frangi vesselness filtering to select the candidate catheter voxels from 3D US, which enables to dramatically reduce the number of samples to be classified by the ConvNet (typically a reduction from $\sim 10^6$ to $\sim 10^4$). From our previous study [4], this simple selection resulted into a high false positive rate because of the weak voxel discrimination in noisy and low-quality cardiac 3D images. To address this, we introduce an adaptive thresholding method for the VOI selection. 3D US images are first filtered by a Frangi filter with a pre-defined scale and rescaled to the unit interval [0, 1], so-called ${\mathcal {V}}$. After the filtering, we apply an adaptive thresholding method to ${\mathcal {V}}$ to coarsely select *N* voxels with the highest vesselness response. The thresholding method is trying to find out the top *N* possible voxels in ${\mathcal {V}}$. Because the filter response has a large variance in different images, the adaptive tuning of the threshold can gradually select *N* voxels, by iteratively increasing or decreasing the threshold *T* based on the image itself. The pseudocode is described by Algorithm 1. Based on the pre-selected voxels in 3D US with remaining voxels being around *N*, the 3D patches are extracted and processed to generate the three orthogonal slices of each voxel for the ConvNet. In our experiment, the initial threshold is empirically set to be $T=0.3$. Value *N* is empirically selected to balance the efficiency of ConvNet classification and classification performance. More details are shown in “Voxel-of-interest selection” section.

### Voxel classification by ConvNet

**Fig. 2:**
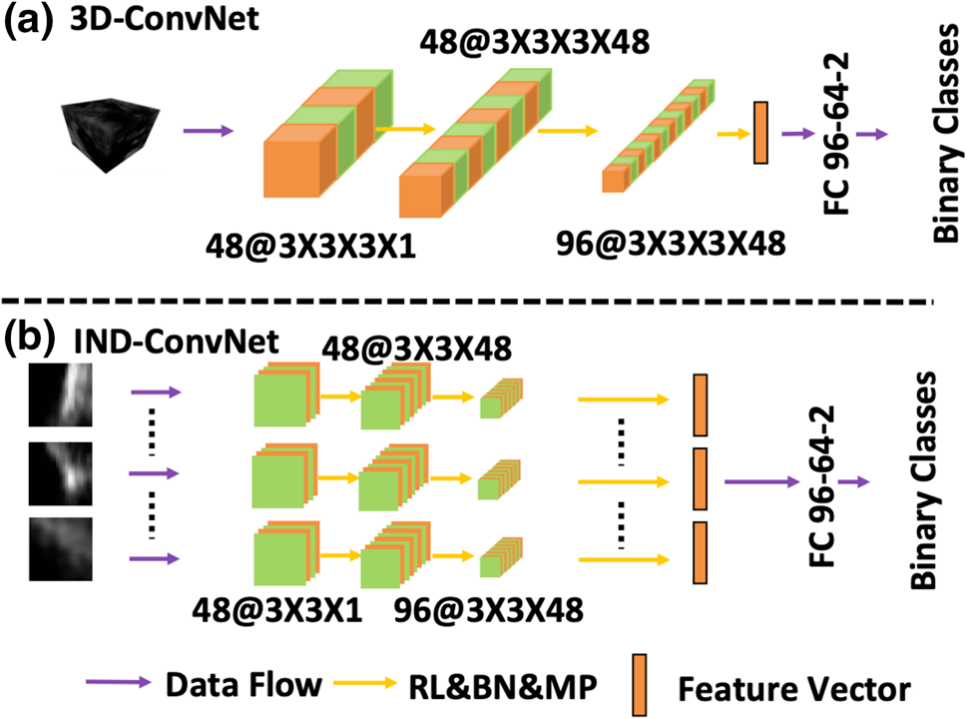
The configurations of commonly used ConvNets. **a** 3D-ConvNet, **b** IND-ConvNet (Note: IND can have branches more than three)

For voxel-wise classification of volumetric data, the 3D local information is processed by ConvNet to classify the voxels. The straightforward way is to classify the voxel based on its 3D neighborhoods. For each candidate voxel located at the center of a 3D cube, the cube is processed by a 3D-ConvNet [6], as shown in Fig. 2a. However, when using a 3D data cube as input, this approach includes too many parameters in the network, which hampers the efficiency of the voxel-wise classification in 3D US volumes. To preserve the 3D information and yet reduce the convolution operations, especially going from 3D operation to 2D operation, the multi-slice-based method was proposed in [11]. To keep the 3D structure information, [11] employed a multi-view cross-section method, which extracts slices from the 3D cube through different angles. Then, each slice will be processed by an individual ConvNet. An example of this method is shown in Fig. 2b, which is called IND-ConvNet. The extracted feature vectors from the slices are concatenated to feed them into fully connected layers (FCs). As for 3D-ConvNet, it processes the information using 3D operations, which leads to too many computations and large execution times. As for IND-ConvNet, although it keeps 3D information by a slicing approach, multiple individual ConvNet branches lead to redundancy, which comes from using a ConvNet for each slice. Because of these redundancies in the networks, 3D-ConvNet and IND-ConvNet are sub-optimal choices in terms of application and computation time.

**Fig. 3:**
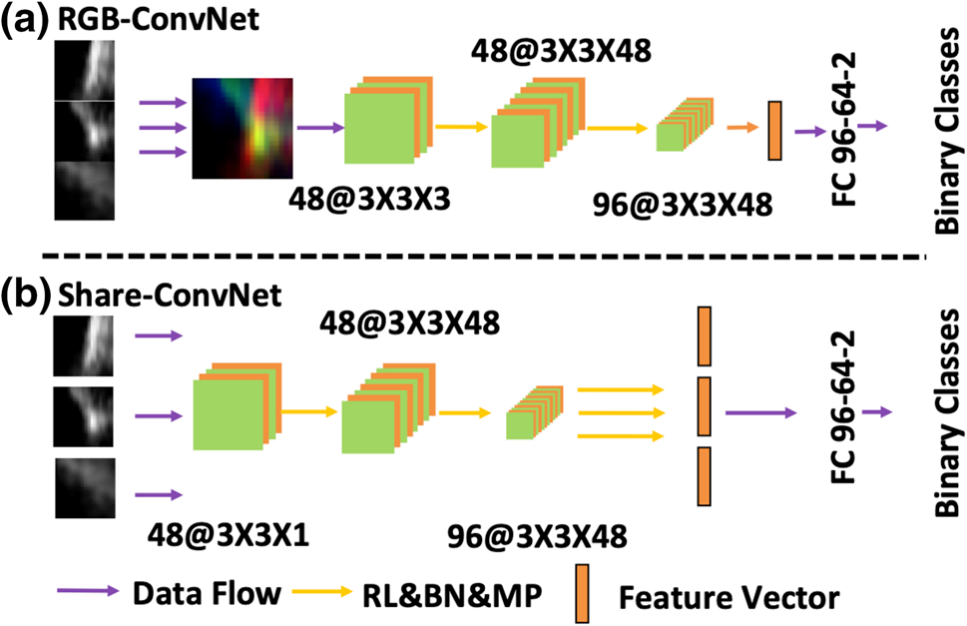
Simplified ConvNets. **a** RGB-ConvNet, **b** Share-ConvNet

**Fig. 4:**
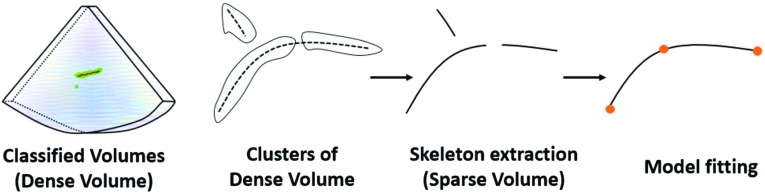
Steps of SPD-RANSAC model fitting

In this work, we attempt to propose a simplified method to classify the voxels. We follow the slice-based strategy. Instead of training ConvNet for each slice, we propose to train one shared ConvNet for all slices. All feature vectors from the shared ConvNets are concatenated to form a longer feature vector for classification. We call it Share-ConvNet, which is shown in Fig. 3b. There is a similar structure called RGB-ConvNet [7], as shown in Fig. 3a. It extracts three orthogonal slices from the principal directions of the 3D cube, which are then reorganized into RGB channels. However, this introduces a limitation: the spatial information between each slice is processed rigidly by convolutional filters at the first stage of the network. With shallow processing by ConvNet, only low-level features are processed and this simple strategy cannot fully exploit the spatial relationships. Alternatively, our proposed Share-ConvNet can exploit the spatial correlation in high-level feature space. Based on the binary selection of candidate voxels during the pre-selection, a 3D cube is obtained for each candidate voxel located at the center of the cube. We extract a cube of size $25\times {25}\times {25}$ voxels, which is larger than a typical catheter diameter of 4–6 voxels in 3D cardiac US. Then, three orthogonal planes passing through the center point of the cube are sliced as the input for the ConvNet (Fig. 3b).

For training with medical images, the class imbalance is the most challenging issue. In our case, the ratio of catheter voxels vs. non-catheter voxels is commonly less than 1/1000. As a consequence and to fully exploit image information, we perform a two-step training when training the ConvNets. First, the number of imbalanced voxels in training images are re-sampled on non-catheter voxels to obtain the same amount as catheter voxels. These balanced samples train the ConvNets. Then, the training images are validated on the trained models to select the falsely classified voxels, which are used to update the networks for finer optimization [8, 9]. Specifically, unlike the diagram in Fig. 1, the training process is applied in the whole US image rather than the VOI processed one. This update step reduces the class imbalance by dropping out the easiest sample points (so-called two-stage training). The parameters of networks are learned by minimizing the cross-entropy, using the Adam optimizer for faster convergence. During the two-step training, the cross-entropy is characterized into a different form to balance the class distribution. In the first training stage, the cross-entropy is characterized in a standard format. However, during the updating, the function is redefined as weighted cross-entropy. These different entropies avoid the bias in the updating stage, which occurs due to the number of false positives being usually 5 to 10 times larger than the positive training samples in the second stage. As a result of the weighted cross-entropy, the networks tend to preserve more catheter voxels than discarding them after the classification. The weighted cross-entropy is formulated in Eq. (1), where the *y* indicates the label of the sample, while ${\hat{p}}$ is the class probability of the sample, and parameter *w* is the sample class ratio among the training samples. During the training, the dropout is used to avoid overfitting with 50% probability in FCs together with an L2 regularization with $10^{-5}$ strength. The initial learning rate is set to be 0.001 and rescaled by a factor 0.2 after every 5 epochs. Meanwhile, to generalize the network in orientation and image intensity variation, data augmentation techniques like rotation, mirroring, contrast and brightness transformations are additionally applied. The mini-batch size is 128, and the total training epoch is 20 which are around 25k in the first training, while iterations in the second training are around 100k.

1 $$\begin{aligned} {\mathrm{Loss}(y,{\hat{p}})=-(1-w){y}{\mathrm{log}({\hat{p}})}-w(1-y){\mathrm{log}(1-{\hat{p}})}.}\nonumber \\ \end{aligned}$$

### Catheter localization

The classified volume may include some outliers, which are generated from the blurry tissue boundaries or catheter-like anatomical structures. To robustly localize the catheter, we employ our previously designed SPD-RANSAC method to fit a pre-defined catheter model [12]. A curved cylinder models the catheter with a fixed radius, which is set to be three voxels in this paper. To robustly localize the catheter, the classified volume, so-called dense volume, is processed by connectivity analysis to generate clusters. Then, the cluster skeletons are extracted to generate the sparse volume. During the fitting stage, three control points are automatically and randomly selected from the sparse domain and ordered in orientation by principal components analysis. The reordered points ensure the cubic spline fitting passes the points in sequential order, which generates the catheter-model skeleton which is shown in Fig. 4. The localized skeleton with the highest number of inliers in the dense volume is adopted as the fitted catheter. The inliers are determined by its Euclidean distances to the skeleton.

**Fig. 5:**
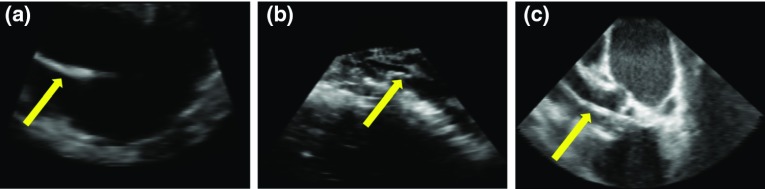
Appearance of different dataset. **a** Phantom US, **b** Pig Heart US, **c** Human Heart. US arrays are pointing to the catheters

**Table 1:** Characterization of 3D ultrasound volumes for experiments

Dataset	Catheterdiameter (mm)	Volume number	Probe type andfrequency range (MHz)	Spatial sizeper voxel (mm)	Volume size (lat.$\times$az.$\times$ax.)
Ex vivo 1	2.3^†^	10	TEE 2-7	0.4	$179\times {175}\times {92}$
Ex vivo 2	2.3^‡^	33	TEE 2-7	0.4	$174\times {174}\times {178}$ to $197\times {197}\times {202}$
Ex vivo 3	2.3^‡^	10	TEE 2-7	0.6	$120\times {69}\times {92}$ to $193\times {284}\times {190}$
Ex vivo 4	2.3^§^	12	TTE 1-5	0.7	$137\times {130}\times {122}$

†Available from Chilli II, ‡Available from Biosense, §Available from OSYPKA

## Datasets and experimental results

### Datasets

In this study, we have collected 4 ex vivo datasets on 4 isolated pig hearts, which resemble the human heart (1 heart for 1 dataset). Table 1 summarizes these datasets. Dataset 1 was collected with a Philips CX-50 machine, while the rest was collected by a Philips EPIQ-7 US machine. Dataset 4 was recorded by TTE (Transthoracic Echocardiogram), which explains the larger voxel size, while other datasets were recorded by TEE (Transesophageal Echocardiography). During the recording, all the images were tuned to have the best visual perception. However, due to equipment variations, the US parameters were different for each dataset. Moreover, to make sure the images in each dataset are independent from each other, we changed the relative position between the heart and US probe to obtain a different appearance of the heart in each captured image. Furthermore, we extracted the catheter and re-inserted it into the heart chambers to make the images independent, i.e., 1 session for 1 image. All datasets were re-sampled to obtain an isotropic spatial resolution and were annotated manually by experts. Examples of three cases are shown in Fig. 5, which compares the recordings on phantom heart, pig heart and human heart. Compared to the phantom heart and human heart, the captured pig heart images are more complex. Compared to the phantom data, real pig tissue has more complex anatomical structures, which makes it hard to distinguish between the catheter and tissue. When compared to the real human heart, the chambers of the pig heart are collapsed due to the dead tissue, which leads to a small free space within the heart. Moreover, the human heart image, which is shown here, has a larger field-of-view than the pig heart recordings, as the data were collected for Transcatheter Aortic Valve Implantation (TAVI) operation. To fully make use of the limited datasets for deep learning, we performed threefold cross-validation on all collected images.

**Fig. 6:**
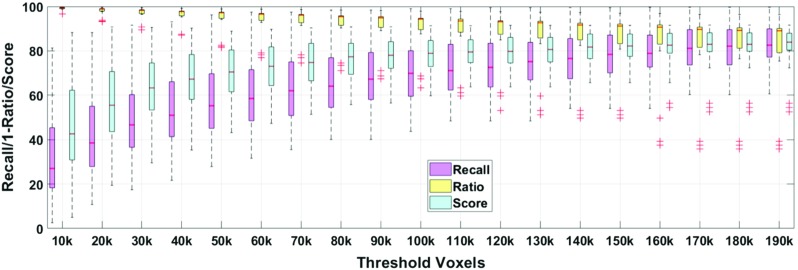
Performance of adaptive thresholding

### Voxel-of-interest selection

To reduce the number of voxels for classification, we applied the Frangi vesselness filter to select the candidate voxels. However, it cannot filter out the catheter voxels from tissue and background with a pre-defined scale due to too many false positives [4]. In our method, we first apply the Frangi filter with scale size equal to 2.5 to filter out the most tubular-like structures. Then, Frangi responses are rescaled to the unity interval, which maps response into a probability-like range.

To evaluate the performance of thresholding, we employ three metrics: Recall (the remaining catheter voxels versus ground-truth catheter voxels), Ratio (thresholded voxels versus all voxels, to evaluate the voxel preserving ability) and their fusion score (mimic $F_1$ score by replacing Precision by Ratio to evaluate a joint threshold performance), which enables to show the preservation performance of catheter voxels and removes non-catheter voxels. The metrics are defined in Eq. (2), where TP is true positive, FN is false negative, TV is remaining voxels after the threshold, while AV is all voxels, giving the specification:

2 $$\begin{aligned} \mathrm{Recall}= & {} \frac{\mathrm{TP}}{\mathrm{TP}+\mathrm{FN}},\nonumber \\ \mathrm{Ratio}= & {} \frac{\mathrm{TV}}{\mathrm{AV}},\nonumber \\ \mathrm{Score}= & {} \frac{2\cdot {\mathrm{Recall}}\cdot {(1-\mathrm{Ratio})}}{\mathrm{Recall}+(1-\mathrm{Ratio})}. \end{aligned}$$

The performances of adaptive thresholding are shown in Fig. 6, where the voxel threshold *N* ranges from 10k to 190k voxels with step size 10k. The values are obtained by averaging of all the testing volumes through threefold cross-validation. It can be observed that the adaptive thresholding method provides a more stable voxel distribution, i.e., a smaller fraction of the whole pyramid area while keeping a higher recall. As a result, the proposed thresholding method provides a better selection for Voxel-of-Interest. However, this pre-selection leads to a drop in Recall. As a consequence, in the following step, a ConvNet with high Recall for voxel classification is needed.

**Table 2:** Average performance of voxel-based classification ($\hbox {mean}\pm \hbox {std.}$)

Method	Recall (%)	Precision (%)	$F_2$ score (%)
GF-SVM [3]	$29.9\pm {25.4}$	$9.2\pm {8.8}$	$19.0\pm {15.5}$
MF-AdaB [4]	$61.2\pm {17.6}$	$28.4\pm {16.6}$	$45.5\pm {15.6}$
3D-UNet [10]	$30.3\pm 26.3$	$11.9\pm 12.7$	$21.4\pm 19.5$
Share-ConvNet	$72.3\pm {19.6}$	$46.4\pm {8.5}$	$63.8\pm {14.3}$

### Voxel classification

#### Comparison with existing methods

**Fig. 7:**
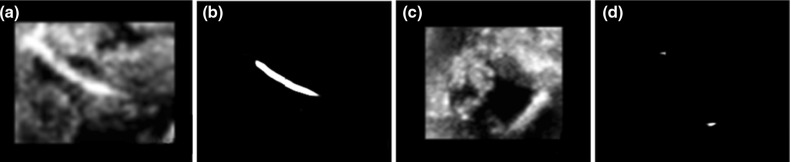
Segmentation results from 3D-UNet. **a**, **b** Successful segmentation and its original image, **c**, **d** failed segmentation and its original image

In the following experiments, three metrics, Recall, Precision and $F_2$ score, are used for voxel classification at image level and defined in Eq. (3) (FP is false positive). We first compare Share-ConvNet with the start-of-the-art methods. Two methods using handcrafted features, Gabor feature with SVM (GF-SVM) [3] and multi-scale and multi-definition features with Adaboosting (MF-AdaB) [4] are considered as baseline. We also consider the semantic segmentation method 3D-UNet [10]. The performances are shown in Table 2. We can see that the Share-ConvNet outperforms conventional methods with handcrafted features. The standard 3D UNet also produces the worst performance on our challenging data. This might be due to 3D UNet being much more complex, resulting in overfitting. Figure 7 shows some example results of 3D UNet.

3 $$\begin{aligned}&\mathrm{Recall}=\frac{\mathrm{TP}}{\mathrm{TP}+\mathrm{FN}},\nonumber \\&\mathrm{Precision}=\frac{\mathrm{TP}}{\mathrm{TP}+\mathrm{FP}},\nonumber \\&F_2=\frac{5\cdot {\mathrm{Recall}}\cdot {\mathrm{Precision}}}{4\cdot {\mathrm{Precision}}+\mathrm{Recall}}. \end{aligned}$$

**Fig. 8:**
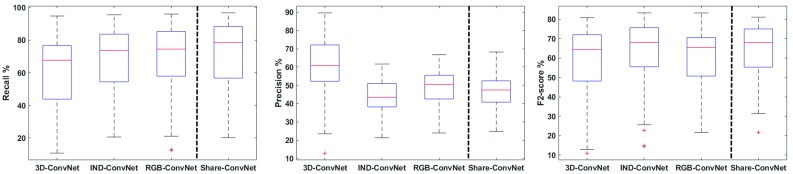
Boxplots of the performance comparison under different metrics. 3D-ConvNet, IND-ConvNet, and RGB-ConvNet are shown at the left side of the dashed line, while Share-ConvNet is shown at right side of the dashed line

#### Comparison with different ConvNet methods

We further compare the Share-ConvNet with 3D-ConvNet, IND-ConvNet, and RGB-ConvNet. The training strategy of these ConvNets is the same as Share-ConvNet. The performance comparison is shown in Fig. 8.

- When compared to 3D-ConvNet, our Share-ConvNet has better Recall and higher $F_2$ score, while 3D-ConvNet achieves better precision. However, taking 3D data cubes as input, 3D-ConvNet has too many parameters in the network, requiring a large amount of training data. In contrast, the Share-ConvNet is much simpler. In terms of efficiency, 3D-ConvNet executes in about 10 min. per volume on average, which is almost $5\times$ more than the orthogonal slice approaches.
- The IND-ConvNet, which is designed to have multiple branches, delivers comparable performance as the proposed Share-ConvNet, since both networks fuse the high-level information in a similar intuition. However, the IND-ConvNet trains an individual ConvNet for each slice, which is computationally complex and leads to redundancy.
- Compared to RGB-ConvNet, we can observe that the Share-ConvNet achieves consistently better performance. It can be explained by that the spatial correlation among different slices in RGB-ConvNet is combined in a lower feature space.

**Table 3:** Paired *t* test (*p* value) between different methods

Method	MS-AdaB	RGB-ConvNet	IND-ConvNet	3D-ConvNet
Share-ConvNet	3.2e−14	3.2e−6	0.26	4.4e−3

**Table 4:** Ablation studies on proposed Share-ConvNet ($\hbox {mean}\pm \hbox {std.}$)

Method	Recall (%)	Precision (%)	$F_2$ score (%)
Share-ConvNet-NoBoost	$92.4\pm 8.6$	$12.0\pm 8.5$	$35.2\pm 17.2$
Share-ConvNet-NoWeight	$45.5\pm 20.9$	$71.3\pm 13.7$	$47.6\pm 20.4$
Share-ConvNet-Combine	$72.3\pm 19.6$	$46.4\pm 8.5$	$63.8\pm 14.3$

#### Paired *t* test between methods

We take the F2 score of each image as a measure and conduct paired *t* tests between our Share-ConvNet with other voxel-based classification methods, i.e., MS-AdaB, RGB-ConvNet, IND-ConvNet, and 3D-ConvNet. In our paired *t* tests, the significance level is set to 0.05. The detailed *p* values for the paired *t* tests are shown in Table 3. All *p* values are smaller than 0.05 except with IND-ConvNet, which shows the Share-ConvNet performs significantly better than MF-AdaB, RGB-ConvNet and 3D-ConvNet methods. Although IND-ConvNet showed little difference with Share-ConvNet, it has parameter redundancy that leads to overfitting and computational inefficiency.

**Table 5:** Comparison of ConvNets with/without VOI ($\hbox {mean}\pm \hbox {std.}$)

Method	Recall (%)	Precision (%)	$F_2$score (%)	Time (s)
VOI-90k-IND-ConvNet	$53.3\pm 17.7$	$58.8\pm 11.7$	$53.4\pm 15.3$	$6.9\pm 0.4$
VOI-190k-IND-ConvNet	$62.6\pm 19.2$	$52.6\pm 10.7$	$59.2\pm 15.9$	$15.1\pm 1.3$
IND-ConvNet	$69.8\pm {20.1}$	$47.7\pm {11.0}$	$62.8\pm {16.1}$	$110.5\pm 59.0$
VOI-90k-Share-ConvNet	$53.7\pm 16.4$	$59.1\pm 11.0$	$53.9\pm 13.9$	$6.5\pm 0.4$
VOI-190k-Share-ConvNet	$63.1\pm 17.8$	$53.0\pm 10.0$	$59.8\pm 14.1$	$14.1\pm 1.2$
Share-ConvNet	$72.3\pm {19.6}$	$46.4\pm {8.5}$	$63.8\pm {14.3}$	$103.4\pm 55.7$

**Table 6:** Performance comparison on catheter localization

Method	EE (mm)	SE (mm)	VS (%)	AHD (voxel)
MF-AdaB	$3.33\pm 2.76$	$2.91\pm 2.55$	$67.3\pm 20.7$	$6.71\pm 7.72$
Share-ConvNet	$2.25\pm 1.91$	$1.83\pm 1.28$	$76.7\pm 13.5$	$1.72\pm 1.85$
VOI-90k-Share-ConvNet	$2.07\pm 1.22$	$1.71\pm 1.00$	$77.3\pm 11.6$	$1.56\pm 2.32$
VOI-190k-Share-ConvNet	$2.08\pm 1.22$	$1.73\pm 0.99$	$77.8\pm 11.6$	$1.64\pm 1.82$

*EE* End-point error, *SE* Skeleton-point error, *VS* Volumetric similarity, *AHD* Average Hausdorff distance

#### Ablation study of ConvNets

The Share-ConvNet includes two-stage training and a weighted loss function in the network. To better understand their influence on the classification performance, we performed ablation studies in three different cases: (1) ConvNet without two-stage training (No Boost), i.e., only trained on re-sampled images, (2) ConvNet with two-stage training but without weighted loss function (No Weight), (3) the proposed ConvNet (Combine). The results of ablation studies are shown in Table 4. As for Share-ConvNet-NoBoost, although it receives relatively high Recall performance, the simple sampling strategy leads to worse Precision results which makes the model fitting more challenging. Furthermore, the weighted loss function can re-balance the information distribution during second-stage training and can maintain a high recall while omitting the non-catheter voxels. When compared with the no-weighted case, the weighted function provides less variance in false positive voxels and $F_2$ scores. In the following experiments, we will consider ConvNet-Combine as the standard network architecture.

#### Share-ConvNet combined with VOI selection

Table 5 compares the performance of ConvNet with or without VOI, where different *N* values (adaptive thresholding to control the voxel cardinality) are considered. When sacrificing voxel cardinality size (fewer voxels), the benefit is a reduced computational complexity, e.g., going from $\sim$100 s processing time to $\sim$10 s per volume, where the VOI selection is still able to reduce the number of false positives at the cost of a slight drop in $F_2$ score (for larger *N*). Although the VOI selection degrades the system performance, it dramatically decreases the number of voxels to be classified by ConvNet. For comparison, IND-ConvNet is also included in the table, which shows a small performance degradation in efficiency and accuracy (with/without VOI selection). Moreover, IND-ConvNet also has more parameters in the model and is therefore more complex than Share-ConvNet. The time was measured on a Titan 1080Ti GPU.

### Catheter localization

Based on voxel classification, the model fitting is applied to the binary images to localize the catheter (its skeleton and end-points) and remove the outliers. We employ the following metrics to measure the model fitting performance: skeleton-based metrics, Volumetric Similarity (VS) and Average Hausdorff Distance (AHD) [13]. More specifically, skeleton-based metrics include two specific types: (1) end-points error (EE) characterized by the average distance between corresponding end-points on the detected catheter and the end-points on the annotation; (2) skeleton error (SE): the average distance between 5 equally sampled points on the detected skeleton and the ground-truth skeleton. Skeleton error has more robust performance than EE. This performance difference is explained by analyzing the difficult cases. For example, sometimes the catheter tip is attaching to the tissue so that it is hard to distinguish the tip from the tissue in B-mode imaging, as shown in Fig. 7a. In such case, the EE metric will give a higher error but SE has inherently better accuracy. However, EE would be more informative than SE, because correctly localizing the tip of the catheter can facilitate the success of the intervention.

We here compare the catheter localization performance based on MF-AdaB, Share-ConvNet, VOI-90k-Share-ConvNet, and VOI-190k-Share-ConvNet. The localization performances are shown in Table 6, which are the average of threefold cross-validation with five times fitting in each volume. The table shows that our proposed Share-ConvNet method achieves a better performance with a lower position error, smaller than the diameter of the catheter. Furthermore, the results show that the VOI-based ConvNet can boost the localization precision in terms of the lowest error. When comparing the results in Tables 5 and 6, VOI provides lower $F_2$ score, but better localization accuracy. This is because VOI provides a higher Precision performance so that a better sparse volume can be achieved. The model fitting relies on the SPD model fitting where fewer outliers would make randomly control points selection more stable. Moreover, with a sacrifice of $F_2$ score through VOI selection, we achieved $10 \times$ faster voxel-based classification, which has shown the trade-off between classification accuracy and efficiency. The whole chain based on VOI-90k-Share-ConvNet takes around 10 s (Frangi filtering: 1.5 s, VOI selection: 0.3 s, ConvNet: 6.8 s and SPD-RANSAC: 1.9 s).

## Conclusion

We have presented an automated catheter localization method using ConvNet. We propose a VOI-pre-selection to reduce the computation load for voxel classification significantly. We have compared different ConvNet methods for voxel classification. Based on the classified voxels, our method can localize the catheter with an average end-point error of about 2.1 mm while executing in 10 s per volume. In future work, we will validate our method on more clinical datasets. Moreover, the speed of 10 s per volume is still far from the real-time performance required in clinical practice, so we have to further improve the efficiency.
